# Temporal trends in heart failure over 11 years in the aging Korean population: A retrospective study using the national health insurance database

**DOI:** 10.1371/journal.pone.0279541

**Published:** 2022-12-28

**Authors:** Dong-Hyuk Cho, Chan Joo Lee, Jung-Woo Son, Jimi Choi, Jinseub Hwang, Byung-Su Yoo

**Affiliations:** 1 Division of Cardiology, Department of Internal Medicine, Korea University, Seoul, South Korea; 2 Division of Cardiology, Department of Internal Medicine, Severance Hospital, Yonsei University College of Medicine, Seoul, South Korea; 3 Division of Cardiology, Department of Internal Medicine, Yonsei University, Wonju College of Medicine, Seoul, South Korea; 4 Division of Endocrinology and Metabolism, Department of Internal Medicine, Korea University College of Medicine, Seoul, South Korea; 5 Department of Computer Science and Statistics, Daegu University, Gyeongsangbuk-do, South Korea; UAB Hospital, UNITED STATES

## Abstract

**Background:**

Understanding national trends of heart failure (HF) is crucial for establishing prevention and treatment strategies. We aimed to investigate the 11-year trends of HF in the South Korean population.

**Methods:**

Using the Korean National Health Insurance Service database, we identified 3,446,256 patients with HF between 2004 and 2014.

**Results:**

The prevalence of HF was 1.42% in 2004, steadily increasing to 1.98% in 2014. However, the age-adjusted prevalence of HF remained stable (1.43% in 2014). The incidence of HF was 6.1/1000 person-years in 2004 and remained at similar levels, reaching 5.4/1000 person-years in 2014. The age-adjusted incidence of HF slowly decreased to 3.94/1000 person-years in 2014. The event rate for hospitalized patients with HF remained stable increasing from 1.40 in 2004 to 1.87/1000 person-years in 2014, and the age-adjusted event rate of hospitalized HF decreased to 1.22 in 2014.

**Conclusions:**

In South Korea, between 2004 and 2014, the prevalence of HF increased while the incidence of HF remained stable. Furthermore, the age-adjusted HF prevalence was stable, and the age-adjusted incidence decreased. This indicates that the aging population is the main cause of the increasing national burden associated with HF and that further attention is warranted in the management of HF in older adults.

## Introduction

Developments in the treatment strategies for heart failure (HF) have improved HF related clinical outcomes; however, HF remains a socioeconomic burden with high mortality and readmission rates [1, 2]. There are currently over 6.5 million patients with HF in the United States, with 1 million new patients with HF added each year, and this situation is similar in most developed countries [3]. Notably, owing to aging of the general population and the increase in associated comorbidities, the number of patients with HF continues to increase worldwide [4]. Furthermore, recent economic developments have promoted excessive nutritional intake as well as sedentary lifestyles, which have led to an increase in cardiovascular risk factors, leading to HF [5]. The increasing rates of HF prevalence and hospitalization are not only a considerable economic burden on patients and families but also have an impact on the broader community [6]. Therefore, nationwide and contemporary epidemiology studies of HF are warranted to establish preventative strategies and inform national resource planning.

In most countries, the number of patients with HF continues to increase, and in the context of the increase in the population over the age of 65 years, the incidence of HF is expected to increase further [4, 7]. However, there are limited data on the estimated prevalence and incidence of HF and its temporal trends, even in developed countries, and these are inconsistent [1, 8–10]. Furthermore, previous studies included selected cohorts or sample populations. The Korean National Health Insurance Service (NHIS) covers the entire Korean population, and the NHIS database contains medical information about those insured. Therefore, the purpose of this study was to analyze the epidemiology of HF and understand the prevalence, incidence, hospitalization rate, and comorbidities across a long period of time, specifically between 2004 and 2014, using the NHIS database of the entire South Korean population.

## Methods

This study used data from the entire NHIS database. The details of the NHIS in the Republic of Korea have been described previously [11]. Briefly, the NHIS is an obligatory social insurance system that covers universal medical healthcare for the population of South Korea. All medical institutions, including private clinics and hospitals, and public health centers submit claims for the reimbursement of covered costs. The claims include not only medical records of inpatient and outpatient care, including diagnostic codes, examination codes, prescription records, and procedure codes, but also personal information. The diagnostic codes are based on the 10th revision of the International Classification of Diseases (ICD-10) [12]. This study used data from the NHIS database spanning 2004–2014. We selected patients with a diagnosis of HF (ICD-10 codes I11.0, I50, I97.1). For the accuracy of diagnosis, we only included patients with one inpatient claim or at least two outpatient claims for HF. The requirement for written informed consent was waived because the NHIS data contain strictly anonymized clinical data that follow the guidelines of the Personal Data Protection Act. This study was approved by the Institutional Review Board (institutional review board number: CR316020), according to the principles of the Declaration of Helsinki.

Comorbidities and outcomes were identified from diagnostic codes in the claims data. The definitions of each disease were based on those from a previously reported protocol [13]. Patients with comorbidities were defined as those with hypertension (ICD-10 codes I10-13 and I15), diabetes (ICD-10 codes E10-14), dyslipidemia (ICD-10 code E78), ischemic heart disease (ICD-10 codes I21, I22, I25.2 or percutaneous coronary intervention or coronary bypass graft), myocardial infarction (ICD-10 codes I21, I22, I25.2), atrial fibrillation (ICD-10 code I48), ischemic stroke (ICD-10 codes I63, 64), hemorrhagic stroke (ICD-10 codes I60-62), chronic kidney disease (eGFR < 60 mL/min per 1.73 m^2^), malignancies (ICD-10 codes C00-97), and osteoporosis (ICD-10 codes M80-82, except for M82.0). To ensure accuracy, comorbidities were established based on one inpatient or two outpatient records of ICD-10 codes for each disease. Hospitalized HF was defined as an admission with diagnostic codes of HF and the intravenous use of diuretics. Medical facilities were divided into tertiary hospitals, general hospitals, hospitals, and clinics according to the Korean Medical Service Act.

### Statistical analyses

Baseline characteristics are presented as numbers with percentages for categorical variables. Data were stratified by year of diagnosis, age, sex, type of hospital, and comorbidities. The year-specific prevalence was calculated by dividing the number of patients with HF by the number of total population-year in the cohort and was presented as percentages. The year-specific incidence and event rates were calculated by dividing the number of incident cases of HF by total population-year and were presented for 1000 person-years. We calculated the age-standardized prevalence, incidence, and hospitalization rates to adjust for age over time. The age-standardized rate is a weighted average of the age-specific rates per 1,000 persons, where the weights are the proportions of persons in the corresponding age groups of the 2004 South Korean population. Data were analyzed using SAS version 9.4 software (SAS Institute, Cary, NC, USA).

## Results

### The prevalence of heart failure

**Fig 1:**
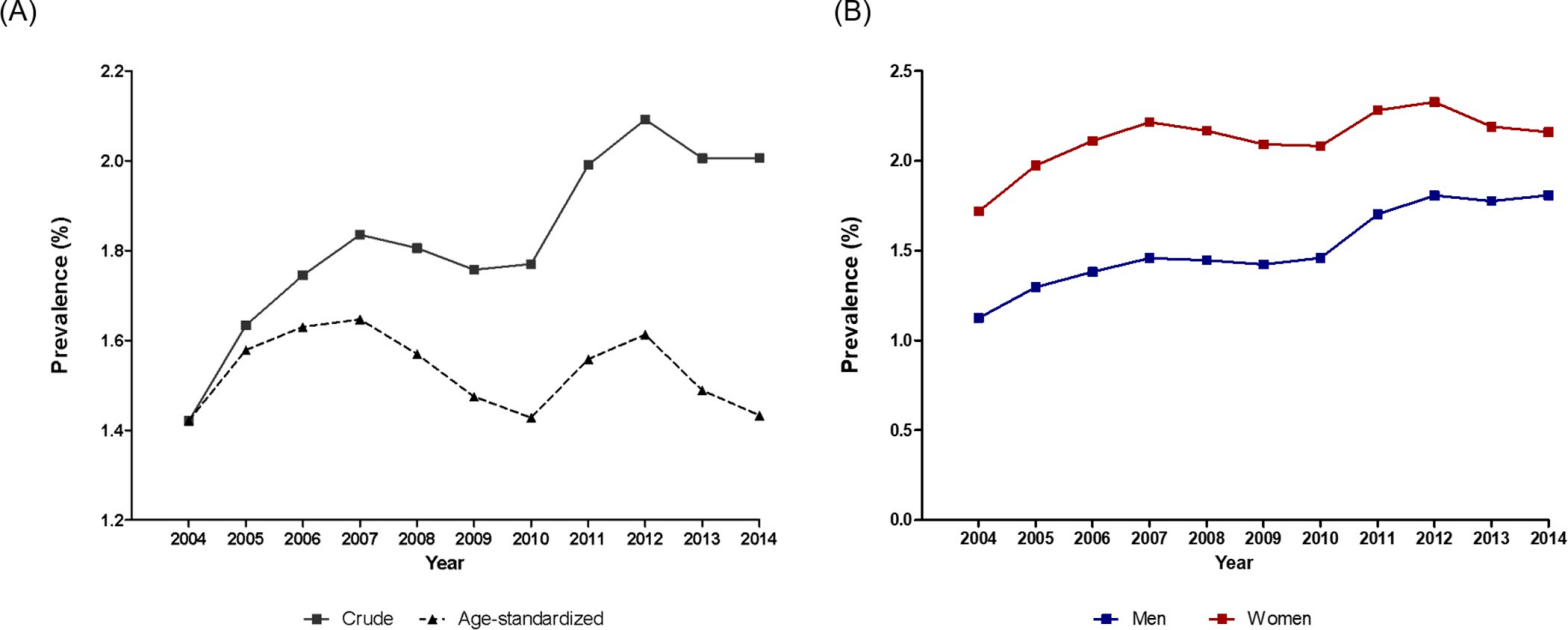
Prevalence of HF in (**A**) total population and (**B**) men and women between 2004 and 2014. The crude prevalence of HF was 1.42% in 2004 and steadily increased to 1.98% in 2014. However, the age-adjusted prevalence of HF tended to be stable at 1.42% in 2004, increasing to 1.43% in 2014. The prevalence rates of HF in men and women was 1.1% and 1.7%, respectively, in 2004, while that in women was consistently higher than that in men over the 11-year study period (1.8% and 2.1%, respectively, in 2014).

In South Korea, the number of patients with HF was 690,219 in 2004, increasing to 1,018,336 in 2014. The prevalence of HF between 2004 and 2014 is shown in Fig 1. The crude prevalence of HF was 1.42% in 2004 and steadily increased to 1.98% in 2014. However, the age-adjusted prevalence of HF was 1.42% in 2004 and remained stable at 1.43% in 2014. The prevalence rates of HF in men and women were 1.1% and 1.7% respectively, in 2004, and the prevalence of HF in men and women was 1.1% and 1.7%, respectively, in 2004, while that in women was consistently higher than that in men over the 11-year study period (1.8% and 2.1%, respectively, in 2014).

**Fig 2:**
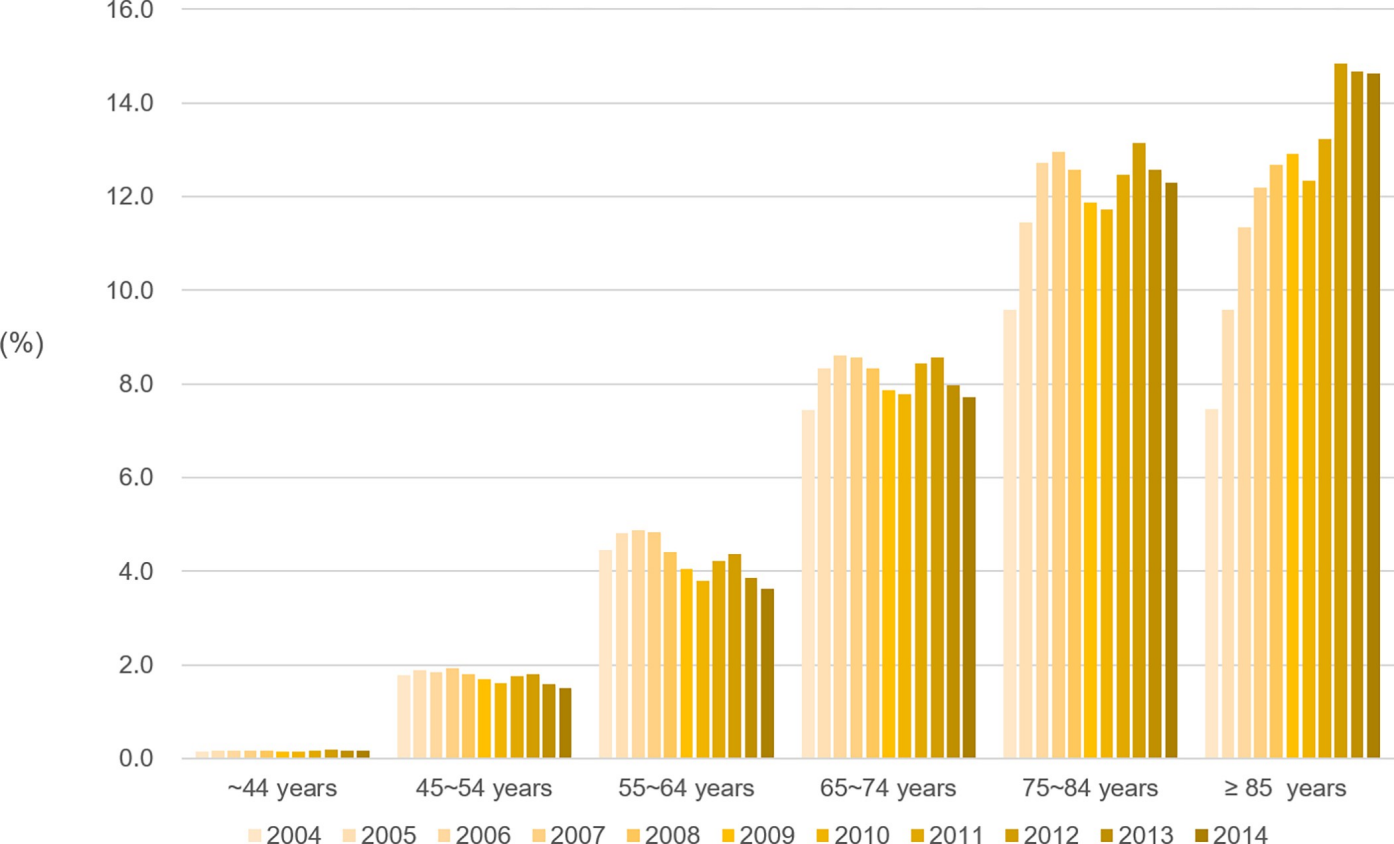
Prevalence of HF according to the age group between 2004 and 2014. The prevalence of HF was steeply increased especially in older adults (over 85 years old).

The prevalence of HF by age is shown in Fig 2. The prevalence of HF increased with age, and this trend was observed until 2014. Furthermore, the prevalence of HF in older people aged ≥75 years increased steadily. In individuals aged 75–84 years and those ≥85 years old, the prevalence of HF was 9.58% and 7.47%, respectively, in 2004, with a 1.3- and 2.0-fold increase to 12.30% and 14.62%, respectively, in 2014. In 2004, the prevalence of HF was the highest in individuals aged 75–84 years. Starting from 2008, we observed a steep increase in the prevalence of HF in older adults aged ≥85 years, which became the highest prevalence across the different age populations. These findings suggest that the age of onset for HF significantly increased within the study period.

### The incidence of heart failure

**Fig 3:**
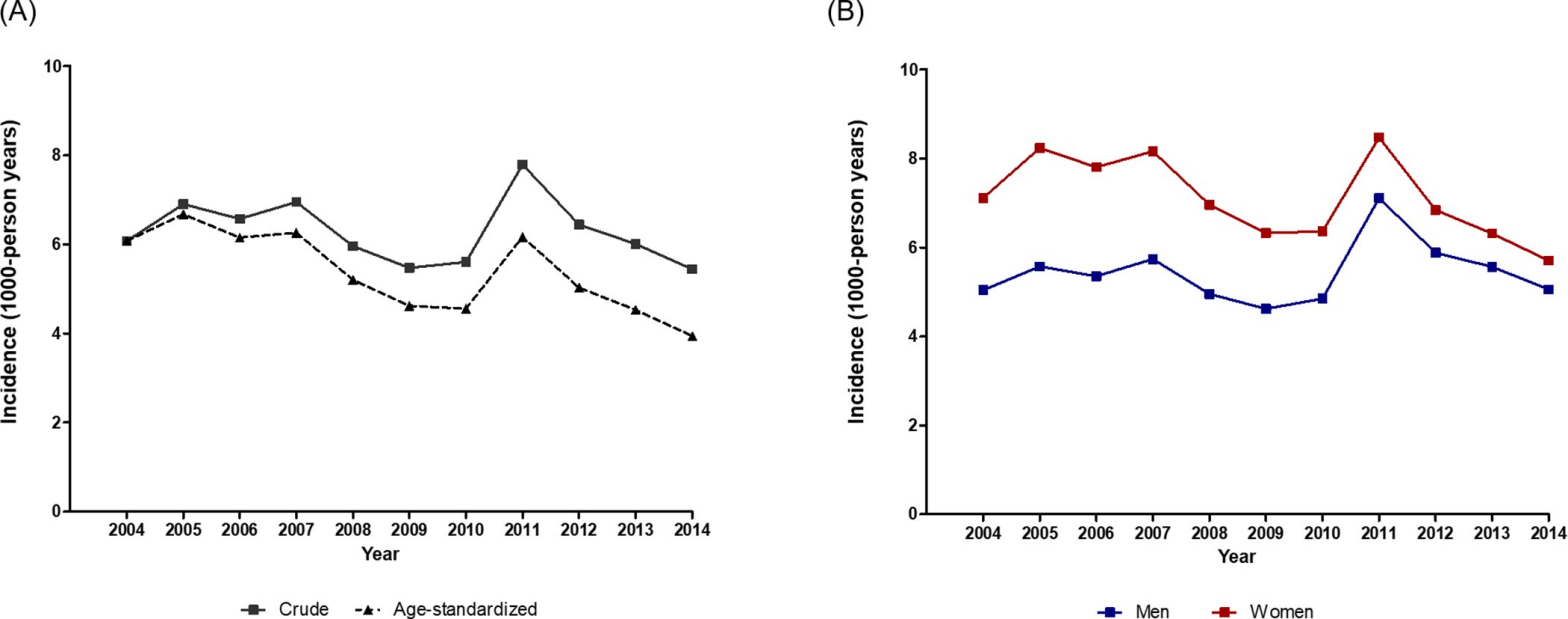
Incidence of HF in (**A**) total population and (**B**) men and women between 2004 and 2014. The crude incidence of HF was stable between 2004 and 2014. The age-adjusted incidence of HF decreased from 6.07 per 1000 person-years in 2004 to 3.94 per 1000 person-years in 2014.

Between 2004 and 2014, HF occurred in 3,445,256 patients. Each year, approximately 300,000 patients were newly diagnosed with HF. The crude incidence of HF was 6.07 per 1000 person-years in 2004 and remained at similar levels, reaching 5.44 per 1000 person-years in 2014 (Fig 3). However, the age-adjusted incidence of HF slowly decreased from 6.07 per 1000 person-years in 2004 to 3.94 per 1000 person-years in 2014. The incidence of HF was higher among women than among men throughout the 11-year study period. The incidence of HF declined among women between 2004 and 2014, although it remained higher than the incidence among men. In 2004, 42% of patients with incident HF were men, although the gap between men and women narrowed by 2014, as 47% of incident HF cases were reported in men.

### Clinical characteristics of patients with incident HF

The temporal trend of clinical characteristics of patients with incident HF between 2004 and 2014 is presented in Table 1. In 2004, the most common chronic diseases among newly diagnosed patients with HF included hypertension (49%), diabetes (20%), and dyslipidemia (16%). Between 2004 and 2014, the proportion of all comorbidities with HF increased, which is likely to be due to the aging HF population. Of those newly diagnosed with HF in 2014, 80% had hypertension, 49% had diabetes, and 66% had dyslipidemia. In 2014, 12% had coronary artery disease, and approximately 21% had cerebral infarction (ischemic stroke and hemorrhagic stroke). The associated malignancy rate also increased from 3.2% in 2004 to 12.7% in 2014. In 2004, most patients with HF (56%) were treated in clinics, and only 9.6% and 23.3% of patients with HF were treated in tertiary and general hospitals, respectively. The proportion of patients with HF treated in tertiary and general hospitals continuously increased to 20.5% and 39.6%, respectively, in 2014. However, only 30.9% of patients with HF were treated in clinics in 2014.

**Table 1:** Temporal trend of clinical characteristics of patients with incident heart failure.

Year	2004	2006	2008	2010	2012	2014
Number of patients	295115	322117	295138	283026	324211	276249
Age						
20–34	7101 (2.4)	6327 (2.0)	5738 (1.9)	5027 (1.8)	6010 (1.9)	5225 (1.9)
35–44	23044 (7.8)	20565 (6.4)	18537 (6.3)	16075 (5.7)	18479 (5.7)	14340 (5.2)
45–54	54371 (18.4)	54423 (16.9)	51308 (17.4)	45620 (16.1)	50776 (15.7)	38223 (13.8)
55–64	77746 (26.3)	74421 (23.1)	63331 (21.5)	60359 (21.3)	73128 (22.6)	59290 (21.5)
65–74	82939 (28.1)	95450 (29.6)	87440 (29.6)	81042 (28.6)	88895 (27.4)	74028 (26.8)
75–84	42272 (14.3)	58424 (18.1)	54413 (18.4)	58692 (20.7)	68096 (21)	64997 (23.5)
≥85	7642 (2.6)	12507 (3.9)	14371 (4.9)	16211 (5.7)	18827 (5.8)	20146 (7.3)
Sex						
Male	122805 (41.6)	131456 (40.8)	123011 (41.7)	122738 (43.4)	150029 (46.3)	129835 (47)
Female	172310 (58.4)	190661 (59.2)	172127 (58.3)	160288 (56.6)	174182 (53.7)	146414 (53.0)
Type of hospitals						
Tertiary hospitals	28280 (9.6%)	29972 (9.3%)	25813 (8.7%)	36800 (13.0%)	71593 (22.1%)	56746 (20.5%)
General hospitals	68842 (23.3%)	82817 (25.7%)	74218 (25.1%)	78712 (27.8%)	113513 (35.0%)	109261 (39.6%)
Hospitals	32691 (11.1%)	40778 (12.7%)	36304 (12.3%)	35527 (12.6%)	31957 (9.9%)	24864 (9.0%)
Clinics	165302 (56.0%)	168550 (52.3%)	158803 (53.8%)	131987 (46.6%)	107148 (33.0%)	85378 (30.9%)
Comorbidities						
Hypertension	145087 (49.2)	184860 (57.4)	205597 (69.7)	213061 (75.3)	256148 (79)	220340 (79.8)
Diabetes mellitus	58876 (20)	82979 (25.8)	100701 (34.1)	114521 (40.5)	145873 (45)	135230 (49)
Ischemic heart disease	6397 (2.2)	11130 (3.5)	14880 (5.0)	21280 (7.5)	32108 (9.9)	32612 (11.8)
Myocardial infarction	5547 (1.9)	9299 (2.9)	12165 (4.1)	16386 (5.8)	23318 (7.2)	22240 (8.1)
Atrial fibrillation	5308 (1.8)	9373 (2.9)	12463 (4.2)	17441 (6.2)	22778 (7.0)	23431 (8.5)
Ischemic stroke	13707 (4.6)	25053 (7.8)	33716 (11.4)	41816 (14.8)	52883 (16.3)	50164 (18.2)
Hemorrhagic stroke	1783 (0.6)	3293 (1.0)	4244 (1.4)	4983 (1.8)	6567 (2.0)	6287 (2.3)
Chronic renal failure	3218 (1.1)	5109 (1.6)	6950 (2.4)	8777 (3.1)	13428 (4.1)	14161 (5.1)
Malignancies	9390 (3.2)	15489 (4.8)	20305 (6.9)	25614 (9.1)	35234 (10.9)	35153 (12.7)
Osteoporosis	29936 (10.1)	49807 (15.5)	70598 (23.9)	82599 (29.2)	103008 (31.8)	97426 (35.3)

### Hospitalized patients with heart failure

**Fig 4:**
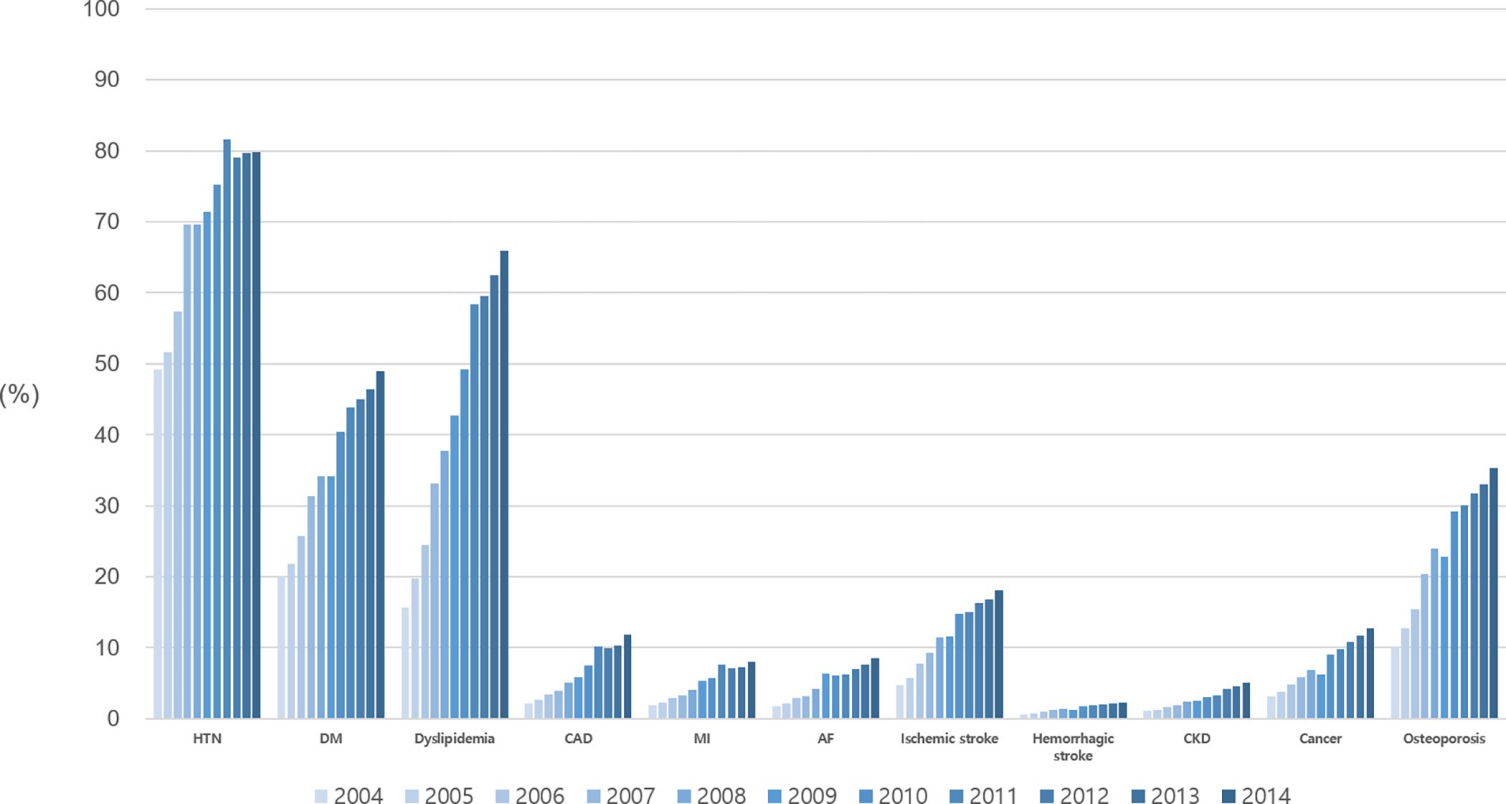
Prevalence of comorbidities in patients with HF between 2004 and 2014. The prevalence of comorbidities in patients with HF increased between 2004 and 2014.

**Fig 5:**
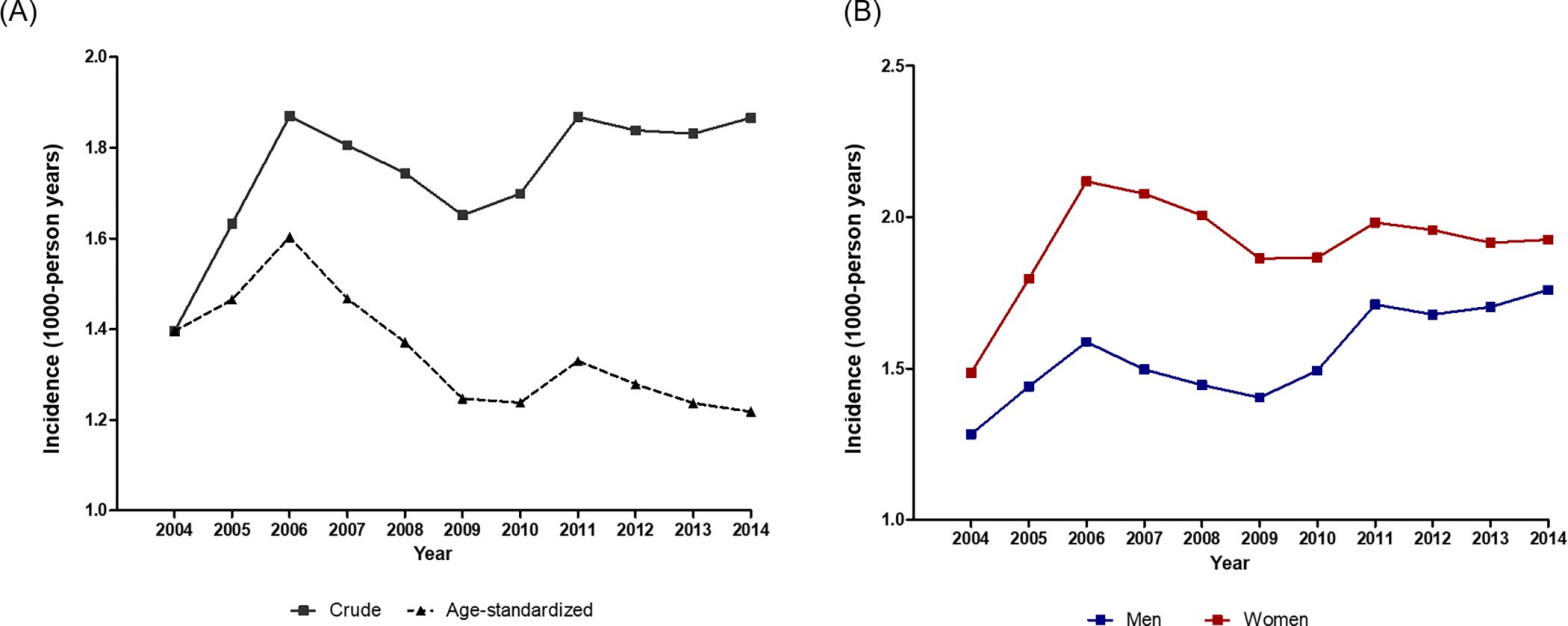
Hospitalized HF (per 1000 person-years) between 2004 and 2014. The number of hospitalized patients with HF increased from 1.40 per 1000 person-years in 2004 to 1.87 per 1000 person-years in 2014.

In 2004, the event rate for hospitalized patients with HF was 1.40 per 1000 person-years. The event rate was higher in women than in men (Fig 4), increasing over the 11-year study period. The event rate of hospitalization for HF increased from 1.40 per 1000 person-years in 2004 to 1.87 per 1000 person-years in 2006 and remained similar until 2014 (Fig 5). In both men and women, the event rate for hospitalized HF slowly increased between 2004 and 2014. However, after adjusting for age, the incidence of hospitalized HF slowly decreased from 1.40 per 1000 person-years to 1.28 per 1000 person-years.

## Discussion

This study is the first study to analyze the data of 3.4 million patients with HF over an 11-year period using the Korean NHIS database. We found that the prevalence of HF in South Korea continued to increase between 2004 and 2014, while the age-adjusted prevalence remained stable. We also found that the incidence of HF was stable and that the age-adjusted incidence slowly decreased. Furthermore, the average age of patients with HF increased, and the age at which individuals developed HF also increased. The major comorbidities such as hypertension, dyslipidemia, and diabetes, also significantly increased. Additionally, we found that the burden associated with hospitalized patients with HF also increased and was predominantly associated with tertiary medical centers. Importantly, a major strength of the current study is that we performed a longitudinal analysis of the age-matched prevalence, incidence, and hospitalization rates of patients with HF across the entire Korean population covering a period of 11 years.

### The national prevalence of HF

In this study, based on the total population of medical insurance subscribers in South Korea, the prevalence of HF was 1.42% in 2004, 1.63% in 2009, and 1.98% in 2014, which corresponds to a 1.5-fold increase in the prevalence of HF over this period. The prevalence of HF in two sampling cohorts of the Korean nationwide registry was estimated as 1.53% and 1.48% respectively, in 2013 [14, 15]. However, these studies sampled only 2% and 25% from the total Korean patient population with HF. The current study which includes the total patient population with HF confirms the estimated prevalence suggested by these earlier studies.

The prevalence of HF continues to increase worldwide. In Europe, the prevalence of HF is reported to be 1.0–2.2%, and it is anticipated that this trend will continue owing to the expansion in the elderly population [3]. In the US, the prevalence of HF is expected to increase by 46% between 2012 and 2030 [16]. In Japan, it was estimated that the number of patients with a reduced ejection fraction would increase from 979,000 in 2005 to 1.3 million by 2030 [17]. Therefore, the increasing prevalence of HF in Korea appears to be consistent with that in other countries.

We found that the age-adjusted prevalence was stable, which is consistent with HF trends in the US between 2006 and 2014 [10]. These findings suggest that in order to understand the national burden of HF, it is important to consider the impact of the aging population. An important finding in our study is that the prevalence of HF continued to increase in patients aged ≥75 years, while it decreased in patients aged <65 years. This is likely due to the fact that the elderly population is growing rapidly in South Korea. The number of individuals aged ≥65 years was 5.45 million in 2010, increasing to 7.07 million by 2017, and is projected to comprise 46.5% of the total population in 2067 [18].

The proportion of patients with HF continuously increased in tertiary and general hospitals from 9.6% and 23.3% in 2004 to 20.5% and 39.6% in 2014, respectively. Simultaneously, the proportion of patients with HF steadily decreased in local clinics from 56.0% in 2004 to 30.9% in 2014. Based on the current real-world data, we cannot identify the precise reason for the increasing number of patients in tertiary or general hospitals. In South Korea, the entire Korean population is covered by the unique Korean NHIS, which guarantees equal access to all types of hospitals. Therefore, patients can choose their preferred type of hospital without any restrictions. In 2011, 15% of outpatient visits of patients with the diagnosis of primary care were in hospitals instead of clinics. This proportion is likely to be high when compared with the rates in other countries [19]. Therefore, patient preference and a lack of restrictions in the Korean NHIS system may have resulted in the increasing number of patients with HF in large-sized hospitals near metropolitan areas. Further research and new health care policies for HF are required for strict stratification and the assignment of the appropriate level of hospital according to the severity of HF.

### The national incidence of HF

We reported that the national incidence of HF based on the total South Korean population was 6.1 and 5.4 per 1000 person-years in 2004 and 2014, respectively. The incidence of HF is closely associated with population age and comorbidities. For example, the pooled analysis from the Framingham Heart Study and Cardiovascular Health Study showed that the incidence of HF between 2000 and 2009 was 18.9 per 1000 person-years, with a mean age of 74 years [20]. A Danish study suggested an incidence rate of 11.5 per 1000 person-years for individuals aged ≥74 years, 3.5 per 1000 person-years for individuals aged 65–74 years, and 1.7 per 1000 person-years for individuals aged 55–64 years [21]. In contrast, the Multi-Ethnic Study of Atherosclerosis (MESA) study which enrolled a population of individuals >45 years of age and excluded those with established cardiovascular diseases, reported a low incidence rate of HF (3.1 per 1000 person-years) [22]. The fact that the study was based on a relatively young population without established cardiovascular diseases could explain the lowest incidence of HF in the MESA population. While these studies reported slightly different incidence rates of HF by age, the findings are consistent and show that the incidence rate of HF increases with age. Our study enrolled the total Korean population aged over 20 years, and patients with underlying cardiovascular diseases were not excluded. The prevalence of ischemic heart disease and myocardial infarction was 11.8% and 8.1%, respectively, in 2014. In our study, the mean age was lower and the prevalence of comorbidity was higher than the data from other patient registries. We consider that our data can be used as a reference representing the real-world burden of HF within a country.

The longitudinal trend of the incidence of HF in Korea also requires further analysis. The crude incidence of HF was stable (6.1/1000 person-years in 2004, reaching 5.4/1000 person-years in 2014). The age-adjusted incidence of HF slowly decreased to 3.94/1000 person-years in 2014. In the United Kingdom, the national incidence also decreased from 3.6 per 1000 person-years in 2002 to 3.3 per 1000 person-years in 2014 [9]. Several reasons may explain the moderate decline in the incidence of HF. Over recent decades, a shift in the paradigm of HF has occurred. The results of major randomized clinical trials supported the neurohormonal theory of HF [23]. The increased use of neurohormonal blockers in the preclinical stages of HF is likely to have reduced the incidence of HF in developed countries. Advanced revascularization strategies and further use of neurohormonal blockers also reduced the progression of myocardial dysfunction in patients post myocardial infarction [9].

### The increasing burden of comorbidities in HF

With the rapid aging of the Korean population, the prevalence of most comorbidities such as hypertension, diabetes, dyslipidemia, and malignancies, has steadily increased. Hypertension is the most common risk factor for HF. A previous cohort study revealed that hypertension antedated the development of HF in 91% of cases and was associated with a 2- to 3-fold higher risk for HF [24]. The most common comorbidity among patients with HF in the current study was hypertension, and it can be assumed that hypertension is an important risk factor for HF. The KorAHF, a South Korean study of the HF registry, reported that the prevalence of hypertension was 62.2%, and another study using the ADHERE-AP, a regional multicenter database of acute decompensated HF in the Asia Pacific, suggested a prevalence of hypertension of 64% [25, 26]. However, these registry data are based on selected populations in specific centers and age groups. In contrast, approximately 80% of patients with HF in this study had hypertension, which may be due to differences in the selection of participants, particularly in view of our focus on older adults. Importantly, the two previous registry studies enrolled patients with acute decompensated HF. The Olmsted country cohort study, which defined the occurrence of HF based on the ICD code, reported that 73.6% of patients with HF with reduced ejection fraction and 89.3% of patients with HF with preserved ejection fraction (HFpEF) in 2008–2010 had hypertension, similar to our findings [27].

In the Olmsted country cohort study, HF combined with diabetes was associated with a poor prognosis [28]. For example, a study including a multinational cohort of 9,428 outpatients with congestive HF enrolled in the European Society of Cardiology and Heart Failure Association Long-Term Registry, found that the prevalence of diabetes was 36.5%, and diabetes increased the risk of 1-year all-cause death, cardiovascular death, and hospitalization for HF by 28%, 28%, and 37%, respectively [29]. The REPORT-HF, a recent study that examined the clinical features of patients with HF, also indicated that the proportion of patients with diabetes among the patients with HF was 30.9–46.7%, depending on the region [7]. We found that the prevalence of diabetes among patients with HF increased to 49% in 2014.

In South Korea, the prevalence of patients with dyslipidemia has been increasing [30]. Similarly, the prevalence of dyslipidemia in patients with HF had continued to increase from 2004 to 2014. Dyslipidemia characterized by high non-HDL cholesterol and low HDL-cholesterol levels is a well-known risk factor for HF independent of myocardial infarction [31]. The components of metabolic syndromes, such as hypertension and dyslipidemia, cause a synergic effect on the systemic inflammation that interferes with endothelium-cardiomyocyte signaling, leading to cardiomyocyte stiffness and hypertrophy, which are considered to be two of the underlying mechanisms of HF [32]. Our findings suggest that the underlying metabolic risk factors are increasing in patients with HF, indicating the need for more aggressive treatment strategies for these common risk factors in order to improve clinical outcomes.

Finally, the increasing prevalence of malignancies (3.2% in 2004, 12.7% in 2014) indicates that the increasing medical concern in the cardio-oncology field is warranted. In addition to population aging, various factors may explain the increased prevalence of malignancies in patients with HF. Over the past decades, diagnostic and treatment strategies have considerably improved in the oncology field, with corresponding improvements in cancer survival rates [33]. Therefore, the number of cancer survivors continues to increase. Cancer treatment such as chemotherapy and radiation is a well-established risk factor for HF [34]. Furthermore, HF and cancer have common risk factors such as socioeconomic level, smoking, obesity, and diet [35].

### Sex difference

Sex differences in HF epidemiology warrant further discussion. In studies from Western countries, the overall lifetime risk of HF is usually similar between men and women [36, 37]. However, the prevalence, incidence, and event rate of hospitalized HF were persistently higher in women than in men, and the gap steadily decreased during the study period. These findings are consistent with the results of a previous 2002–2013 Korean National Sample Cohort-based study, which indicated a higher prevalence of HF in women than in men (1.72% vs. 1.34%, respectively) [14]. The reason for the higher prevalence of HF in Korean women is unclear. However, several reasons could be postulated. First, old age is one of the key risk factors for the development of HF. Life expectancy in Korean women is the highest in the world [38]. Older mean age in Korean women and the decreased age gap between the sexes in the general population could be one reason for the higher prevalence, incidence, and event rate of hospitalized HF in women than in men. Second, HF with ischemic heart disease is more common in men than in women. Clinicians who managed ischemic heart disease may have omitted coding for HF in men. Third, HFpEF is a phenotype that is generally more common in women than in men. Increased awareness of HfpEF and the availability of echocardiography may have contributed to an increased diagnosis of HFpEF in women. Further studies are warranted to investigate the differences between the sexes in the prevalence of types of HF.

### The increasing burden of HF hospitalization

In the current study, the burden of hospitalized HF cases increased from 1.40 per 1000 person-years in 2004 to 1.87 per 1000 person-years in 2014. However, after adjusting for an aging population, the event rate of hospitalized HF did not change, indicating that aging is an essential factor in the increase of hospitalized HF cases. The Atherosclerosis Risk in Communities study indicated a higher event rate of hospitalized HF (20.7 per 1000 white men, 15.2 per 1000 white women, 38.1 per 1000 black men, and 30.5 per 1000 black women) than that in the current study. The age of the study population in the Atherosclerosis Risk in Communities study (≥55 years) may explain the higher incidence of hospitalized HF [39]. According to a study utilizing nationwide data in the United States, the acute hospitalization rate for primary HF has declined from 4.3 to 3.4 per 1000 person-years. Although the study used a nationwide database, the study population was based only on sampled cases from the emergency departments, explaining the lower incidence of hospitalized patients with HF than that in our study [10]. To the best of our knowledge, this is the first study to evaluate the prevalence, incidence, and hospitalization rate of HF in a nationwide population. Further studies are needed to evaluate the epidemiology of HF in other countries based on nationwide databases.

### Limitations

This study has some limitations. First, the data in this study were limited to the clinical information obtained in the available electronic medical records. Therefore, the lack of information on laboratory findings and medical history made it difficult to identify the underlying risks and severity in the patients. The values of left ventricular ejection fraction, which discriminates the type of HF, were unavailable. Second, clinical research utilizing electrical medical records is highly dependent on the accuracy of the clinical coding input by the attending physicians. Further validation studies are needed to correlate the clinical coding input with the diagnosis in real-world practice. Third, we could not accurately assess the reason behind the changes in the prevalence and incidence of HF because the current study was based on real-world medical claims data. Therefore, further multicenter registries of HF with an emphasis on the precise etiology of HF are warranted to explain the longitudinal trends of the prevalence and incidence of HF. Fourth, we analyzed the prevalence and incidence of HF in the total Korean population. Owing to restrictions of access to the Korean NHIS medical records, we were unable to conduct a more advanced analysis to investigate the independent risk factors of HF. The mortality data of patients with HF were unavailable in the current analysis. Further studies are needed to explore the risk factors, clinical outcomes, and prognosis of South Korean patients with HF.

## Conclusion

Between 2004 and 2014, while the prevalence of HF increased, the incidence of HF and hospitalization rates for HF remained stable in South Korea. Importantly, the age-adjusted prevalence of HF was stable, and the age-adjusted incidence decreased. These findings suggest that the aging population is the main cause of the increasing HF-associated burden at the national level and that more attention is warranted in the prevention and management of HF in the older adult population.

## Abbreviations

HF: heart failure
NHIS: National Health Insurance Service
ICD-10: 10th revision of the International Classification of Diseases
